# Supplementary material for: Transcriptome Signatures of Atlantic Salmon—Resistant Phenotypes against Sea Lice Infestation Are Associated with Tissue Repair
Source: Genes (Basel). 2023 Apr 27;14(5):986. doi: 10.3390/genes14050986 (PMC10218584; doi:10.3390/genes14050986)
Supplement: Supplementary file 1 [file genes-14-00986-s001.zip › Supplementary figures_190423.pdf]

Supplementary figures

Figure S1. Atlantic salmon families behavior under *C. rogercresseyi* infestation. A) Atlantic salmon families sea lice burden per fish after eleven days of infestation. B) PCA analysis of Atlantic salmon families transcriptome profile.

Figure S2. KEGG pathway and Biological Process enrichment analysis of genes differentially expressed in the R and S Atlantic salmon families during a sea lice infestation.

Figure S3. GO enrichment analysis for non-synonymous SNPs variation in the R and the S Atlantic salmon families.

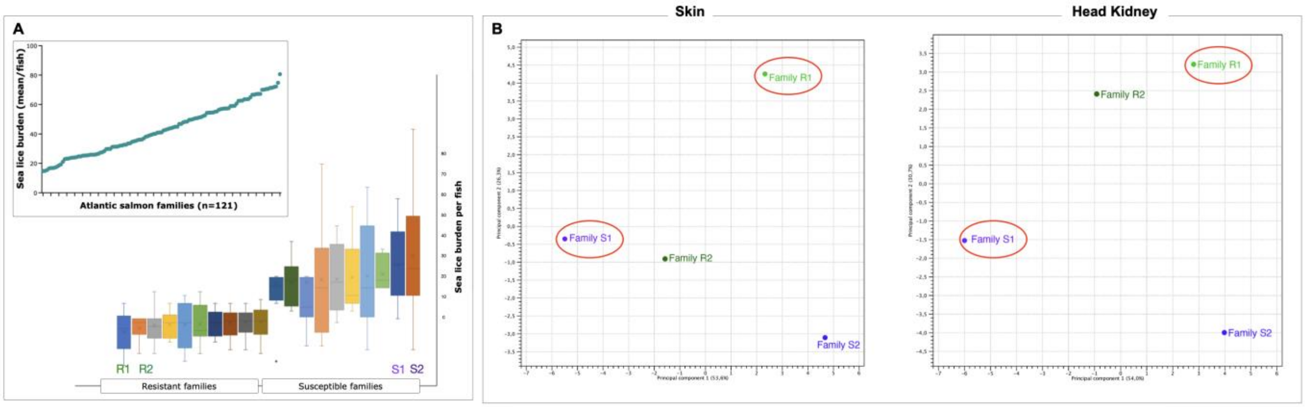

A
Sea lice burden (mean/fish)
Atlantic salmon families (n=121)
Sea lice burden per fish
R1
R2
S1
S2
Resistant families
Susceptible families
B
Skin
Head Kidney
Family R1
Family R2
Family S1
Family S2

Figure S1.

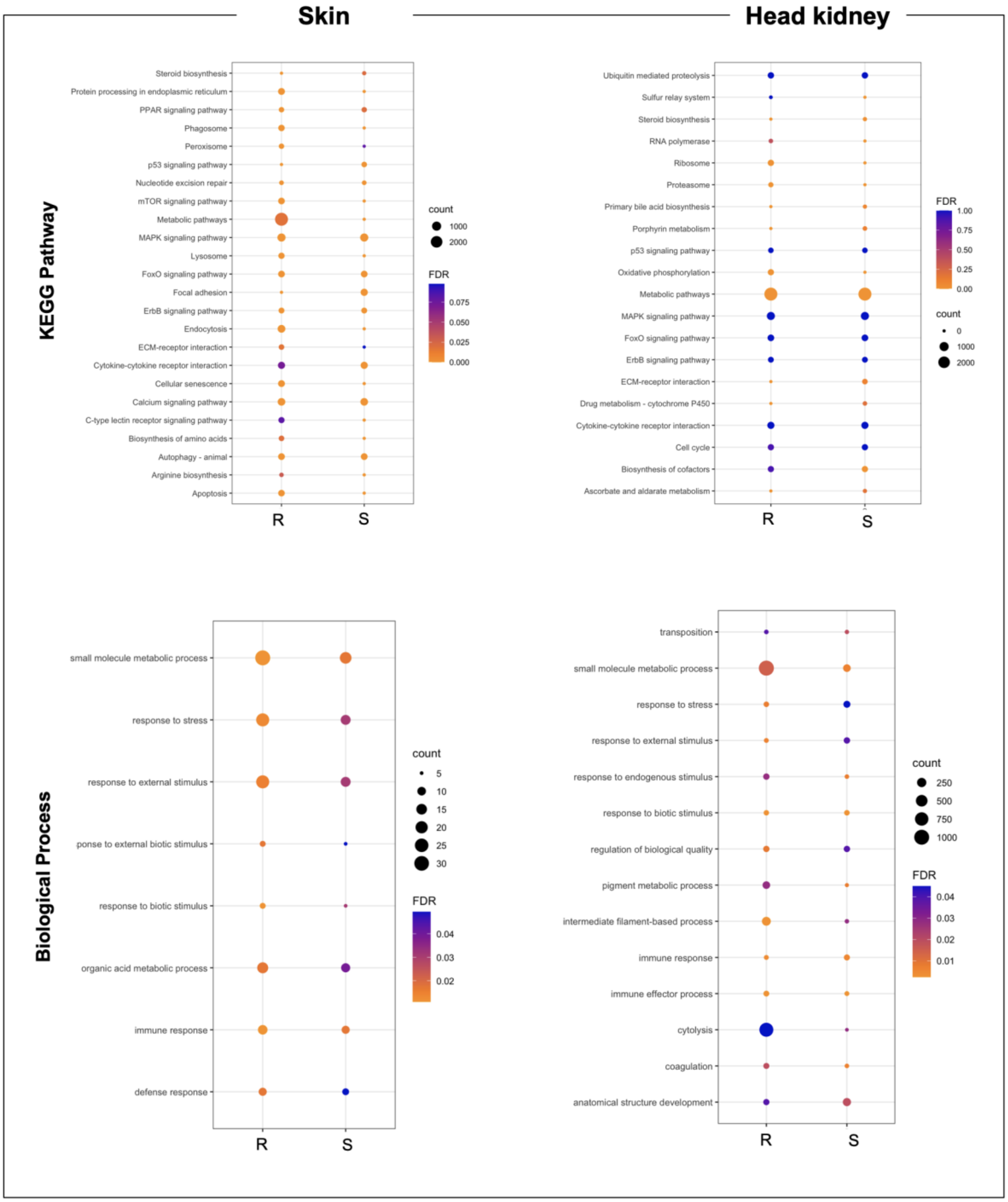

Skin
Head kidney
KEGG Pathway
Steroid biosynthesis
Protein processing in endoplasmic reticulum
PPAR signaling pathway
Phagosome
Peroxisome
p53 signaling pathway
Nucleotide excision repair
mTOR signaling pathway
Metabolic pathways
MAPK signaling pathway
Lysosome
FoxO signaling pathway
Focal adhesion
ErbB signaling pathway
Endocytosis
ECM-receptor interaction
Cytokine-cytokine receptor interaction
Cellular senescence
Calcium signaling pathway
C-type lectin receptor signaling pathway
Biosynthesis of amino acids
Autophagy - animal
Arginine biosynthesis
Apoptosis
R
S
count
1000
2000
FDR
0.075
0.050
0.025
0.000
Ubiquitin mediated proteolysis
Sulfur relay system
Steroid biosynthesis
RNA polymerase
Ribosome
Proteasome
Primary bile acid biosynthesis
Porphyrin metabolism
p53 signaling pathway
Oxidative phosphorylation
Metabolic pathways
MAPK signaling pathway
FoxO signaling pathway
ErbB signaling pathway
ECM-receptor interaction
Drug metabolism - cytochrome P450
Cytokine-cytokine receptor interaction
Cell cycle
Biosynthesis of cofactors
Ascorbate and aldarate metabolism
R
S
FDR
1.00
0.75
0.50
0.25
0.00
count
0
1000
2000
Biological Process
small molecule metabolic process
response to stress
response to external stimulus
ponse to external biotic stimulus
response to biotic stimulus
organic acid metabolic process
immune response
defense response
R
S
count
5
10
15
20
25
30
FDR
0.04
0.03
0.02
transposition
small molecule metabolic process
response to stress
response to external stimulus
response to endogenous stimulus
response to biotic stimulus
regulation of biological quality
pigment metabolic process
intermediate filament-based process
immune response
immune effector process
cytolysis
coagulation
anatomical structure development
R
S
count
250
500
750
1000
FDR
0.04
0.03
0.02
0.01

Figure S2.

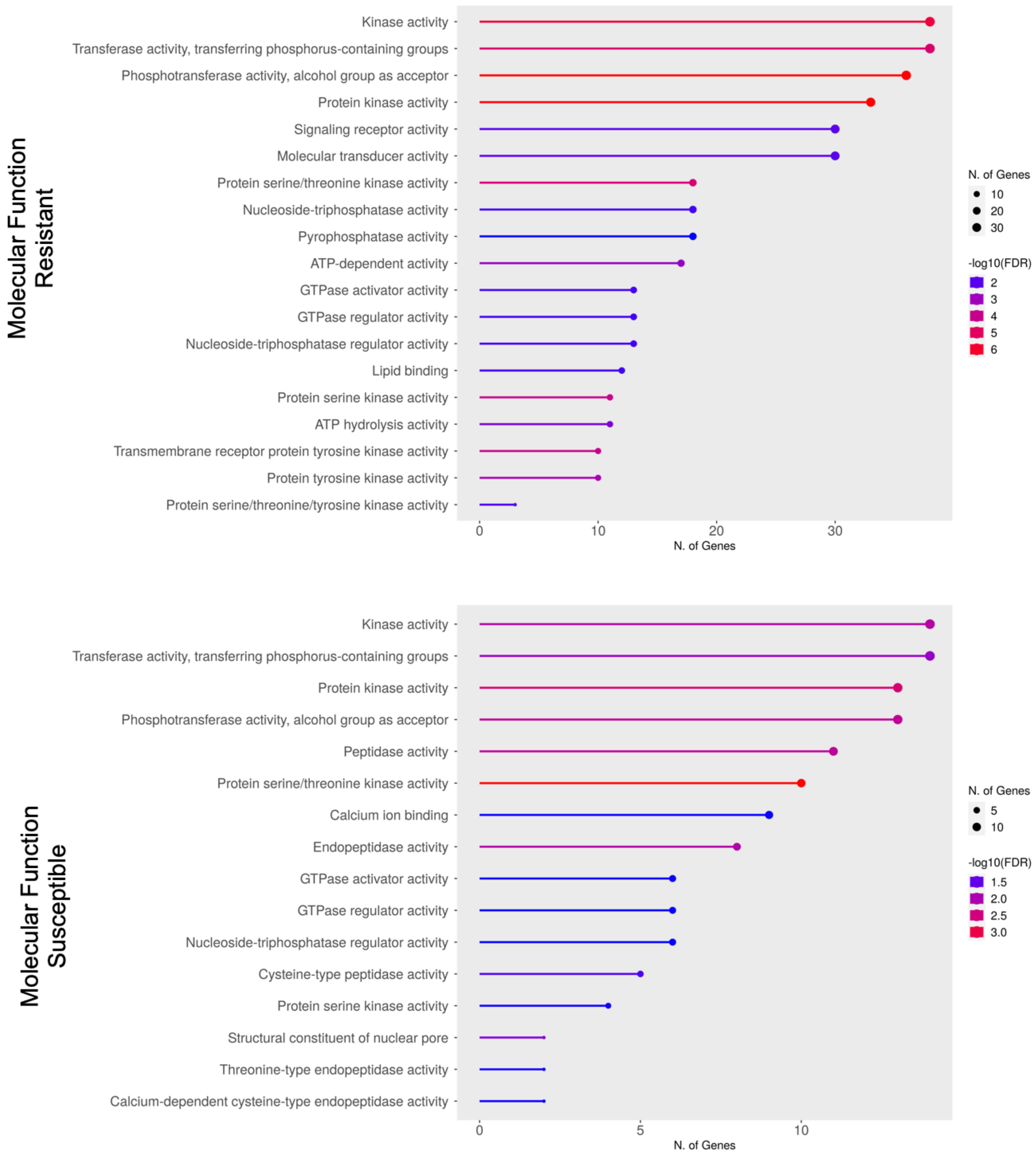

Molecular Function
Resistant
Kinase activity
Transferase activity, transferring phosphorus-containing groups
Phosphotransferase activity, alcohol group as acceptor
Protein kinase activity
Signaling receptor activity
Molecular transducer activity
Protein serine/threonine kinase activity
Nucleoside-triphosphatase activity
Pyrophosphatase activity
ATP-dependent activity
GTPase activator activity
GTPase regulator activity
Nucleoside-triphosphatase regulator activity
Lipid binding
Protein serine kinase activity
ATP hydrolysis activity
Transmembrane receptor protein tyrosine kinase activity
Protein tyrosine kinase activity
Protein serine/threonine/tyrosine kinase activity
0
10
20
30
N. of Genes
N. of Genes
10
20
30
-log10(FDR)
2
3
4
5
6
Molecular Function
Susceptible
Kinase activity
Transferase activity, transferring phosphorus-containing groups
Protein kinase activity
Phosphotransferase activity, alcohol group as acceptor
Peptidase activity
Protein serine/threonine kinase activity
Calcium ion binding
Endopeptidase activity
GTPase activator activity
GTPase regulator activity
Nucleoside-triphosphatase regulator activity
Cysteine-type peptidase activity
Protein serine kinase activity
Structural constituent of nuclear pore
Threonine-type endopeptidase activity
Calcium-dependent cysteine-type endopeptidase activity
0
5
10
N. of Genes
N. of Genes
5
10
-log10(FDR)
1.5
2.0
2.5
3.0

Figure S3.
